# PB.48: Old habits die hard: breast imaging of symptomatic 35 to 39 year olds

**DOI:** 10.1186/bcr3548

**Published:** 2013-11-08

**Authors:** FM Singh, S Gadde

**Affiliations:** 1 Nightingale Centre and Genesis Prevention Centre, University Hospital of South Manchester NHS Foundation Trust, Wythenshawe, Manchester, UK

## Introduction

*The Best Practice Diagnostic Guidelines for Patients Presenting with Breast Symptoms *were published in 2010 and suggest patients younger than 40 years should undergo breast ultrasound (US) as first-line imaging, with additional mammography for indeterminate and suspicious clinical or US findings. However, our current departmental guidelines are to use first-line mammography in patients aged 35 and over. The aims were to quantify the use of imaging modalities and cancer detection; to assess whether additional information gained by mammography changed management; and to quantify how many fewer mammograms would have been performed if we had adhered to the 2010 Guidelines.

## Methods

Imaging and histology reports were reviewed over a 6-month period for 35-year-old to 39-year-old females with a symptomatic breast complaint.

## Results

A total of 247 symptomatic 35 to 39 year olds had breast imaging with a malignancy rate of 4%, all cancers presented with a lump/thickening. Sixty-nine per cent had both US and mammography, 23% mammography only and 9% US only. Four U3 lesions were detected on US with normal mammograms, one of which was malignant. Mammography altered management in three patients, requiring biopsies of the asymptomatic breast, none of which were malignant. If we had adhered to the 2010 Guidelines we would have performed 150 fewer mammograms out of the 171 patients who had both US and mammography.

## Conclusion

In our study, no malignancy would have been missed and we would have performed 150 fewer mammograms if we had adhered to the 2010 Guidelines and used US as a first-line investigation.

